# Rewiring the aging brain: exergaming modulates brain complexity in older adults

**DOI:** 10.3389/fnagi.2025.1748274

**Published:** 2026-01-12

**Authors:** Daghan Piskin, Helen Müller, Nina Skjæret-Maroni, Beatrix Vereijken, Jochen Baumeister

**Affiliations:** 1 Exercise Science and Neuroscience Unit, Department of Sports and Health, Paderborn University, Paderborn, Germany; 2 Department of Neuromedicine and Movement Science, Norwegian University of Science and Technology (NTNU), Trondheim, Norway

**Keywords:** aging, brain complexity, electroencephalography, exergaming, multiscale entropy

## Abstract

**Introduction:**

Age-related changes in brain signal complexity are associated with cognitive decline and reduced neural adaptivity in older adults. Exergaming offers a promising prophylactic intervention combining physical and cognitive training. The aim of the present study was to assess how exergaming alters the temporal trajectory of brain signal complexity at rest and during gameplay in older adults.

**Methods:**

Twenty-eight healthy older adults participated in a 4-week exergaming intervention. Electroencephalography was recorded using 64 electrodes at rest (pre- and post-intervention) and during exergaming (pre-, mid-, and post-intervention). Brain signal complexity was quantified using multiscale entropy across 64 time scales on preprocessed signals.

**Results:**

Post-intervention resting-state analysis revealed significant reductions at fine and increases at coarse scales in frontal, central, and posterior entropy. During gameplay, entropy declined widespread by mid-intervention, particularly at coarse scales over frontal, central and temporal regions. From mid- to post-intervention, the decline narrowed leaving a net pre-to-post reduction concentrated at coarse scales in these regions.

**Discussion:**

Resting-state changes indicated a shift toward a younger brain profile, characterized by a transition from age-related increases in local processing to enhanced distributed processing, which may potentially mitigate the rise in neural modularity associated with aging. During gameplay, brain signal complexity decreased in week 2, followed by a modest change by week 4, consistent with the framework in which complexity initially streamlines and then adjusts toward a task-specific optimum. These findings suggest that exergaming can beneficially modulate brain complexity in older adults, offering the potential to reduce age-related neural decline and support healthy brain aging.

## 1 Introduction

Age-related declines in cognitive and physical functions impose a substantial burden on the independence and wellbeing of older adults, often impeding instrumental activities of daily living and reducing overall quality of life (Deary et al., 2009; Harada et al., 2013). These declines are the manifest outcomes of interacting biological processes that accompany normative aging. A powerful framework for understanding these processes is the “loss-of-complexity” hypothesis, which posits that aging is characterized by a progressive erosion of complex variability across multiple organ systems (Lipsitz, 1992; Lipsitz, 2004). A healthy, youthful physiological system exhibits intricate fluctuations over a wide range of time scales, a feature that enables it to adapt robustly to internal and external perturbations. With age, this rich, structured variability degrades, leading to a more simplified and predictable output that reduces the system’s dynamic range and its ability to respond flexibly to stressors (Sleimen-Malkoun et al., 2014). Within the nervous system, this loss-of-complexity model implies that the rich, multiscale temporal structure of neural activity becomes simplified and less coordinated with age, constraining the brain’s information processing capacity and limiting the repertoire of states available for flexible, adaptive behavior (Takahashi et al., 2009; Cieri et al., 2021). However, “loss-of-complexity” does not imply a uniform reduction at every time scale in this context. Rather, it signifies a specific degradation of coordinated structure across multiple temporal scales, reflecting a breakdown in the efficient interplay between local neural computations and global network integration (McIntosh et al., 2014).

Electroencephalography (EEG) offers a direct window into neural dynamics at the millisecond resolution required to capture these complex temporal patterns. Among entropy-based metrics, Multiscale Entropy (MSE) is well-suited particularly for investigating the loss-of-complexity hypothesis (Costa et al., 2002; Mizuno et al., 2010). Unlike single-scale measures, MSE explicitly evaluates signal complexity from fine to coarse time scales through a coarse-graining procedure (Costa et al., 2002). This method is highly sensitive to age-related alterations characterized by reweighting local, high-frequency processing (fine scales) and distributed, low-frequency integration (coarse scales) (Takahashi et al., 2009; McIntosh et al., 2014; Kosciessa et al., 2020). A robust body of research has characterized a specific pattern of age-related changes in resting-state brain dynamics, showing higher entropy at fine temporal scales and reduced entropy at coarser scales (Sleimen-Malkoun et al., 2015; Wang et al., 2016; Ando et al., 2022; Wan et al., 2023). This pattern is interpreted as a functional shift from efficient, long-range network integration towards a more localized, segregated processing (Vakorin et al., 2011; McIntosh et al., 2014), an alteration indicative of a fundamental disruption of the brain’s integrated network architecture that impairs the ability to flexibly process information across distributed neural systems and ultimately undermines adaptive cognitive function (Fornito et al., 2015). It was shown that higher coarse-scale entropy at rest, particularly in frontal, parietal, and temporal regions, is positively correlated with superior cognitive functioning in older adults, establishing it as a key neural correlate of successful cognitive aging (Ueno et al., 2015; Iinuma et al., 2022).

Given that this age-related decline in resting-state complexity signifies a breakdown in the brain’s integrated network architecture, interventions that may promote long-range communication are of critical importance. Physical exercise has emerged as a promising strategy, inducing physiological and metabolic changes that facilitate cognitive function through brain adaptations (Kirk-Sanchez and McGough, 2013; Xu et al., 2023). Exergaming in particular, which integrates physical exercise with cognitively demanding tasks, represents an enriched evolution of exercise (Donath et al., 2016; Cacciata et al., 2019; Chen et al., 2021). By simultaneously challenging the body and cognition, exergaming offers a potential, multi-modal stimulus for enhanced information integration across brain networks. Its advantage lies in its nature as a form of enriched environmental stimulation, demanding the real-time integration of visuospatial processing, executive functions, and motor control (Guimarães et al., 2021; Ferreira et al., 2024). Studies suggest that this multi-domain integration may potentially induce neuroplasticity, strengthen the complex, long-range neural networks and the information processing capacity of the brain which are compromised in aging (Lungarella and Sporns, 2006; Monteblanco Cavalcante et al., 2021; Henrique et al., 2023; Attoh-Mensah et al., 2025). Still, despite growing interest in exergaming, its neurophysiological effects remain not well established. Current evidence lacks particularly findings with respect to age-related changes in brain complexity.

Previous work has shown that 4-week exergaming interventions can already induce measurable changes in EEG activity and cognitive performance in older adults (Huang, 2020; Müller et al., 2024; Müller et al., 2025). Three recent studies have demonstrated that older adults develop performance strategies with varying game characteristics and difficulty levels, refine their gameplay behavior over time and exhibit increasing cognitive engagement, accompanied by improvements in performance (Müller et al., 2023, 2024, 2025). Accordingly, the primary aim of the current study was to investigate whether a 4-week exergame intervention changes the global resting-state brain complexity across multiple time scales (Ueno et al., 2015; Iinuma et al., 2022). However, an analysis of resting-state dynamics alone provides limited insight into the brain’s capacity to adapt to complex and unpredictable stimuli in real-time. Exergaming challenges an individual’s ability to interact with a dynamic environment, requiring the continuous integration of multimodal cognitive and motor processes. This capacity for effective dual-task performance is known to decline with age and is considered as a sensitive marker of available cognitive resources and executive function (Brustio et al., 2017; Li et al., 2018). While changes in resting-state complexity are well-documented, far less is known about the potential non-linear dynamics that unfold as older adults learn and adapt to a novel, challenging task (Anderson, 1982; Berka et al., 2007). Hence, by assessing brain complexity during gameplay at pre-, mid- and post-intervention points, the current study also aimed to characterize the temporal trajectory of complexity as an index of the cognitive capacity and adaptive processing required to navigate the exergame environment.

## 2 Materials and methods

### 2.1 Participants

Twenty-eight healthy, independently living older adults (14 female; mean age = 74.47 years, range = 70–84 years) were recruited from the local community through advertisements in local newspapers. The study was registered at the German Clinical Trials Register (DRKS00034786, date of registration: 30/07/2024, retrospectively registered) and approved by the Ethics Committee of Paderborn University. All procedures were conducted in accordance with the Declaration of Helsinki and participants provided written informed consent prior to their participation.

Inclusion criteria required participants to be 70 years of age or older and living independently. Individuals were excluded if they had a history of neurodegenerative or major neurologic diseases, acute physical or mental conditions that would prevent safe participation, or recent surgery or injury to the back or lower extremities. Baseline assessments, including the Montreal Cognitive Assessment (MoCA) (Nasreddine et al., 2005) for cognitive function and the Community Balance and Mobility Scale (CBMS) (Howe et al., 2006) for mobility, indicated that the cohort consisted of high-functioning individuals with normal-to-good cognitive and physical functions. The demographic characteristics of the participants are presented in Table 1. The regular use of one or more prescribed medications (e.g., antihypertensives, anticoagulants, diuretics) was common among participants. All 28 participants successfully completed the full 12-session, 4-week intervention protocol.

**TABLE 1:** Demographics of the participants.

	Mean	SD	Min	Max
Age (years)	74.75	0.79	70	84
Height (cm)	172.04	1.93	151	192
Weight (kg)	76.85	2.35	56	102
MOCA	25.04	0.39	21	28

### 2.2 Study design and experimental procedures

The present study was designed as a single-arm pre–post intervention study examining changes in brain signal complexity before and after a 4-week exergame intervention in older adults. The data for the present analysis were drawn from a larger intervention study (Müller et al., 2023, 2024, 2025). The study was conducted and reported in accordance with the TREND (Transparent Reporting of Evaluations with Nonrandomized Designs) guidelines (des Jarlais et al., 2004), and the completed checklist is provided as Supplementary file.

**FIGURE 1:**
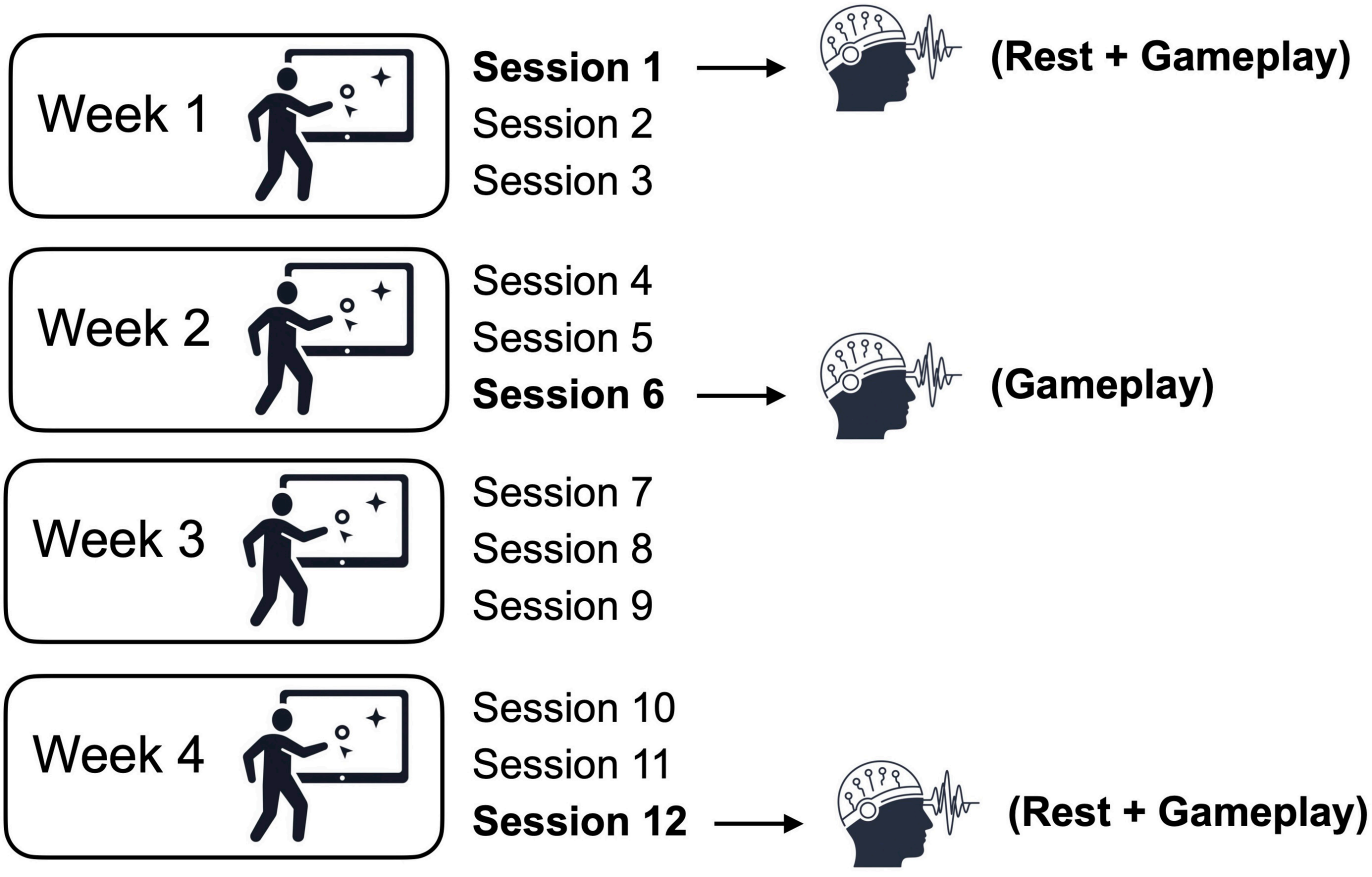
Study design overview. Participants attended weekly training sessions across 4 consecutive weeks. Resting-state EEG recordings were obtained at baseline (session 1) and after completion of the intervention (session 12). EEG recordings during gameplay were collected at pre- (session 1), mid- (session 6), and post-intervention (session 12).

The intervention protocol consisted of 12 training sessions conducted over 4 weeks, with participants attending three sessions per week. Each session lasted approximately 45 min and was conducted on a one-to-one basis with an experimenter at the lab to ensure consistency and provide support. For the present analysis, EEG data from specific appointments were considered. Resting-state EEG data were analyzed from sessions 1 (pre-intervention) and 12 (post-intervention), which had been collected prior to gameplay while the participants sat quietly on a standard chair and looked at a black screen which was set at their eye level. EEG data collected during gameplay were analyzed for session 1 (pre-intervention), session 6 (mid-intervention), and session 12 (Figure 1).

During the training sessions, participants played two distinct exergames (Puzzle and Fox) with the SilverFit 3D system, each at two difficulty levels. The order of these four game conditions was counterbalanced across all participants and sessions to minimize potential order effects. In addition to EEG data, changes in physical function (CBMS) and in-game metrics—including required time, game scores, and game speed level progression—were analyzed by comparing pre- and post-intervention data.

### 2.3 Exergames

The intervention utilized the SilverFit 3D (SilverFit BV, The Netherlands), a virtual rehabilitation system recommended for balance training in older adults (Rademaker et al., 2009). The system uses a TV screen and a time-of-flight camera that records the player’s body movements in three dimensions within a 5 × 5 meter game area, corresponding to a 176 × 144 pixel array.

**FIGURE 2:**
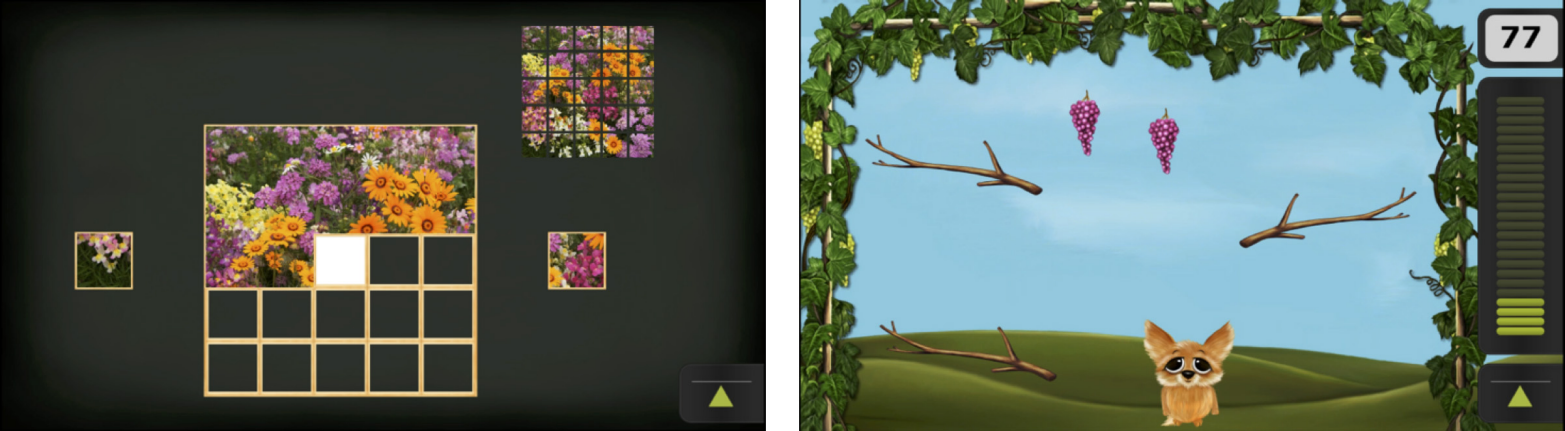
Exergames implemented as intervention. In the Puzzle game (left), participants solved puzzles by moving the correct piece into position through body leaning. In the Fox game (right), participants controlled a fox character by taking steps sideways to avoid obstacles and collect items.

Participants played two different games consisting of leaning (Puzzle) and stepping movements (Fox). The Puzzle game required participants to solve a 5 × 5 piece jigsaw puzzle by leaning their upper body sideways without taking steps (Figure 2). In the easy condition, a single puzzle piece appeared on either the left or right side, and the player selected it by leaning in the corresponding direction. In the hard condition, two puzzle pieces appeared simultaneously, and the player had to select the correct piece by leaning toward it. Performance was scored based on the time taken to solve the puzzle.

The Fox game required participants to take sideways steps to control an on-screen fox avatar to catch falling grapes (Figure 2). In the easy condition, only grapes fell from the top of the screen. In the hard condition, branches also fell, which players needed to avoid while still catching the grapes. To ensure better EEG data quality, participants were instructed to limit arm movements to reduce muscle artifacts. The game score was calculated as the number of grapes caught, with two points deducted for each branch hit. The game speed was individually adapted. All participants started at speed level 3 out of 10. The speed was increased for the next session if a participant caught all grapes without being hit by a branch or rated the game as too slow. Conversely, the speed was reduced if the participant indicated it was too fast. Higher game speed levels involved more grapes appearing and falling at a faster rate.

### 2.4 Behavioral outcomes

Behavioral changes were evaluated using both a standardized measure of mobility and specific in-game metrics. Mobility was assessed pre- and post-intervention using the Community Balance and Mobility Scale (CBMS), which evaluates high-level balance and mobility across 13 distinct tasks, such as tandem walking and lateral dodging (Howe et al., 2006). Each task is scored on a scale from 0 to 5 (one item exceptionally 6), yielding a maximum possible score of 96, where higher scores reflect better functional performance. In-game performance for the Puzzle game was measured by the time required to complete the puzzle, with shorter times indicating better performance. The durations were converted to seconds (s) for analysis. For the Fox game, performance was quantified by a game score, calculated as the number of grapes caught minus a penalty of two points for each branch hit. Additionally, game speed for the Fox game was individually adapted based on performance and subjective feedback, with progression to higher speed levels serving as an indicator of improved performance over the intervention period.

### 2.5 EEG recording and preprocessing

EEG data were recorded continuously using 64 active electrodes (ActiCap, Brain Products, Germany) placed according to the international 10-20 system and a wireless amplifier (Brain Products GmbH, Germany). The sampling rate was 500 Hz, and the reference and ground electrodes were placed at FCz and AFz, respectively (Pivik et al., 1993). Prior to recording, electrode positions were scanned using Cap Track (Brain Products, Germany), and an impedance check confirmed an acceptable signal-to-noise ratio (below 10 kΩ).

Offline preprocessing was conducted using the EEGLAB toolbox (v2025.0) (Delorme and Makeig, 2004) for MATLAB (R2024b, The MathWorks, United States). Based on event markers sent to the data stream during the experiment, continuous data corresponding to the 4-min resting-state and the duration of each gameplay condition were extracted for analysis.

This preprocessing pipeline was adapted from previous studies that applied multiscale entropy analysis on mobile EEG data (Piskin et al., 2025). Sinusoidal line noise was removed from the extracted data using the Cleanline plugin (Mullen, 2012), followed by a finite impulse response (FIR) band-pass filter between 3 and 30 Hz. Noisy channels were automatically identified and rejected based on the following criteria: flatline duration greater than 5 s, high-frequency noise standard deviation greater than 4, and a minimum correlation with nearby channels below 0.8. These rejected channels were then replaced using spherical spline interpolation. After interpolation, the data were re-referenced to the common average and then downsampled to 256 Hz. To clean stereotypical artifacts, an adaptive mixture independent component analysis (Palmer et al., 2011) was applied to decompose the signal into maximally independent components (ICs). Source localization for each IC was performed using the DIPFIT plugin (Oostenveld and Oostendorp, 2002) with a standardized four-shell spherical head model (BESA, Germany). Brain-related ICs were identified based on their spatial localization, a residual variance below 15%, and a classification probability of at least 90% brain activity from the ICLabel plugin (Pion-Tonachini et al., 2019; Piskin et al., 2025). All components not fulfilling these criteria were removed.

### 2.6 Multiscale entropy computation on preprocessed EEG signal

The MSE procedure quantifies signal complexity across multiple time scales and consists of two main steps: a coarse-graining procedure followed by the calculation of sample entropy (SE) for each resulting time series. For reliable entropy estimates, a sufficient number of data points (14*^m^*–23*^m^* for the last scale to be analyzed) is required (Gow et al., 2015). For the resting-state analysis, a continuous segment of 33,856 data points (*m* = 2, 23^2^ * 64 scales: approximately 132 s) was extracted from the beginning of each recording to standardize data length across participants and time points (Piskin et al., 2025).

For the game-related complexity analysis, only data from the Fox game were considered to ensure comparability across conditions. The Fox game featured standardized, continuous stepping dynamics, whereas the Puzzle game involved more variable, self-paced movements that could introduce confounding variability into the complexity estimates. To achieve the target data length of 33,856 points while balancing the contribution of both difficulty levels, the data from the easy and hard conditions of the Fox game were concatenated. Specifically, the initial 16,928 samples from the start of each condition were extracted and merged, creating a single continuous time series for each participant at each measurement point.

MSE analysis was then applied to these standardized, continuous EEG data segments for each channel using a custom MATLAB algorithm. First, for each channel, the time series was coarse-grained across 64 time scales (Iz was excluded leading to 63 channels included in the analysis totally). This procedure creates progressively shorter time series representing the signal’s dynamics at different temporal resolutions by averaging non-overlapping segments of the original signal. The coarse-grained time series y for a given time scale τ is defined as:


$$y_{j}^{T}=1/T+\overset{j\,T}{\underset{i=\left(j-1\right)\,T+1}{\sum }}x_{i},1\leq y_{j}\leq N/T$$

where *x* is the original time series of length *N*, and *j* ranges from 1 to *N*/τ (Costa et al., 2002). This process acts as a low-pass filter, with higher scales progressively capturing lower frequency dynamics.

Next, SE was calculated for each of the 64 coarse-grained time series. SE quantifies the regularity of a time series by measuring the conditional probability that two similar sequences of *m* data points will remain similar when an additional point is included (Richman and Moorman, 2000). The calculation was performed with the following formula:


$$S_{E}\,\left(m,N,r\right)=\ln\frac{A}{B}=\ln\frac{\sum_{i=1}^{N-m}n_{i}^{m}}{\sum_{i=1}^{N-m}n_{i}^{m+1}}$$

An embedding dimension of *m* = 2 and a similarity criterion of *r* = 0.50 × SD, where the standard deviation (SD) was dynamically adjusted for each time scale. This dynamic adjustment of *r* helps prevent a bias toward lower entropy values at coarser scales (Kosciessa et al., 2020). This procedure resulted in a 64 (scales) × 63 (channels) entropy matrix for each participant and condition, which was used for subsequent statistical analysis.

### 2.7 Statistical approaches

All statistical analyses were performed in MATLAB using built-in functions and custom scripts. Distributional assumptions of the behavioral data were evaluated using the Shapiro–Wilk test, visual inspection of histograms and Q–Q plots. Change-score normality was assessed for three contrasts: pre- to mid-, mid- to post-, and pre- to post-intervention. For CBMS, only pre- to post-intervention comparisons were performed. When the relevant change-scores were normally distributed, descriptive statistics were reported as mean ± SD and differences were tested with two-sided paired *t*-tests. Mean differences, their 95% confidence intervals and effect sizes (Cohen’s *d*) were reported. If normality was violated, descriptive statistics were given as median [IQR] and differences were tested with the two-sided Wilcoxon signed-rank test. The median changes were reported with matched-pairs rank-biserial correlations (*r*) as effect sizes. To control the family-wise error rate across these planned comparisons, *p*-values were adjusted using the Holm procedure. Statistical significance was set at α = 0.05 (two-tailed) after adjustment.

Differences in entropy estimates between pre- and post-intervention were assessed with custom scripts using a nonparametric permutation-based framework combined with parametric per-scale paired *t*-tests within the channel. For each participant, channel, and scale (τ), the within-subject change ΔMSE(τ) = Post-Pre was computed. To test whether the scale-wise Δ profiles exhibited structure beyond random fluctuations, the lag-1 autocorrelation (r_1_) of the group-mean Δ profile per channel was quantified and compared to a scale-shuffle null generated by randomly permuting the scale order within participants and recomputing r_1_ (right-tailed permutation test; *N* = 5,000). This adopts the mass-univariate permutation logic for ordered axes commonly used in EEG (Maris and Oostenveld, 2007) and parallels surrogate-based controls used with MSE in electrophysiology (e.g., phase-randomized/IAAFT surrogates) to ensure effects are not explained by linear spectral structure (Schreiber and Schmitz, 2000; Miskovic et al., 2019). For the localization of differences, paired tests of ΔMSE(τ) against zero were performed separately per scale within each channel and multiplicity was controlled within the channels using a false discovery rate (FDR) correction (*q* = 0.05) according to Benjamini and Hochberg (1995), summarizing results as contiguous runs of significant scales. An effect was counted only when ≥ 3 adjacent scales showed significance following FDR. To quantify overall magnitude irrespective of scale, a Complexity Index (CI) per participant was computed as the sum of MSE across scales and the mean ΔCI was tested using a two-sided sign-flip permutation across participants (*N* = 5,000); where relevant, Cohen’s *d* is reported, and permutation *p*-values are reported as *p* < 1/(N+1) when they reach the resolution limit (Schreiber and Schmitz, 2000; Costa et al., 2002; Maris and Oostenveld, 2007; Miskovic et al., 2019).

## 3 Results

### 3.1 Behavioral outcomes

Pre–mid–post analyses showed statistically significant improvements in in-game metrics overall, and CBMS showed a significant pre–post increase. For the Puzzle game, score changes were not significant for pre- to mid- and mid- to post-intervention. However, the pre- to post-intervention increase was significant. Puzzle completion time decreased significantly over both pre- to mid-, mid- to post-, and pre- to post-intervention. For the Fox game, score and speed level increased significantly for pre- to mid-, mid- to post- and pre- to post-intervention. Detailed statistics are provided in Table 2.

**TABLE 2:** Pre–mid-post changes in mobility (CBMS) and in-game metrics with effect sizes.

	Mean ± SD			Pre- to mid-intevention				Mid- to post-intevention				Pre- to post-intevention			
Endpoint	Pre	Mid	Post	Test	Stat	*P*-value (holm adjusted)	Effect size	Test	Stat	*P*-value (holm adjusted)	Effect size	Test	Stat	*P*-value (holm adjusted)	Effect size
CBMS	64.39 ± 10.97	–	68.86 ± 11.85	—	—	—	—	—	—	—	—	PT	*t*(27) = 4.45	0.002	*d* = 0.84
Puzzle score	24.18 ± 0.57	24.37 ± 0.49	24.52 ± 0.31	PT	*t*(27) = 1.99	0.056	*d* = 0.38	WT	*W* = 133	0.173	*r* = 0.42	PT	*t*(27) = 3.47	0.002	*d* = 0.66
Puzzle duration (s)	91.93 ± 10.92	73.32 ± 7.14	68.39 ± 6.75	WT	*W* = 0	< 001	*r* = -1.00	PT	*t*(27) = -3.57	0.001	*d* = -0.68	PT	*t*(27) = -10.85	< 001	*d* = -2.05
Fox score	42.78 ± 7.39	78.03 ± 12.42	87.96 ± 16.83	WT	*W* = 0	< 001	*r* = 1.00	WT	*W* = 28	< .001	*r* = 0.86	WT	*W* = 0	< 001	*r* = 1.00
Fox level	3.09 ± 0.27	4.96 ± 0.58	5.36 ± 0.78	WT	*W* = 0	< 001	*r* = 1.00	WT	*W* = 0	.002	*r* = 1.00	WT	*W* = 0	< 001	*r* = 1.00

Δ = Post - Pre. Effect sizes are given as Cohen’s *d* for paired *t*-tests (PT) and rank-biserial *r* for Wilcoxon tests (WT).

### 3.2 Resting-state multiscale entropy

**FIGURE 3:**
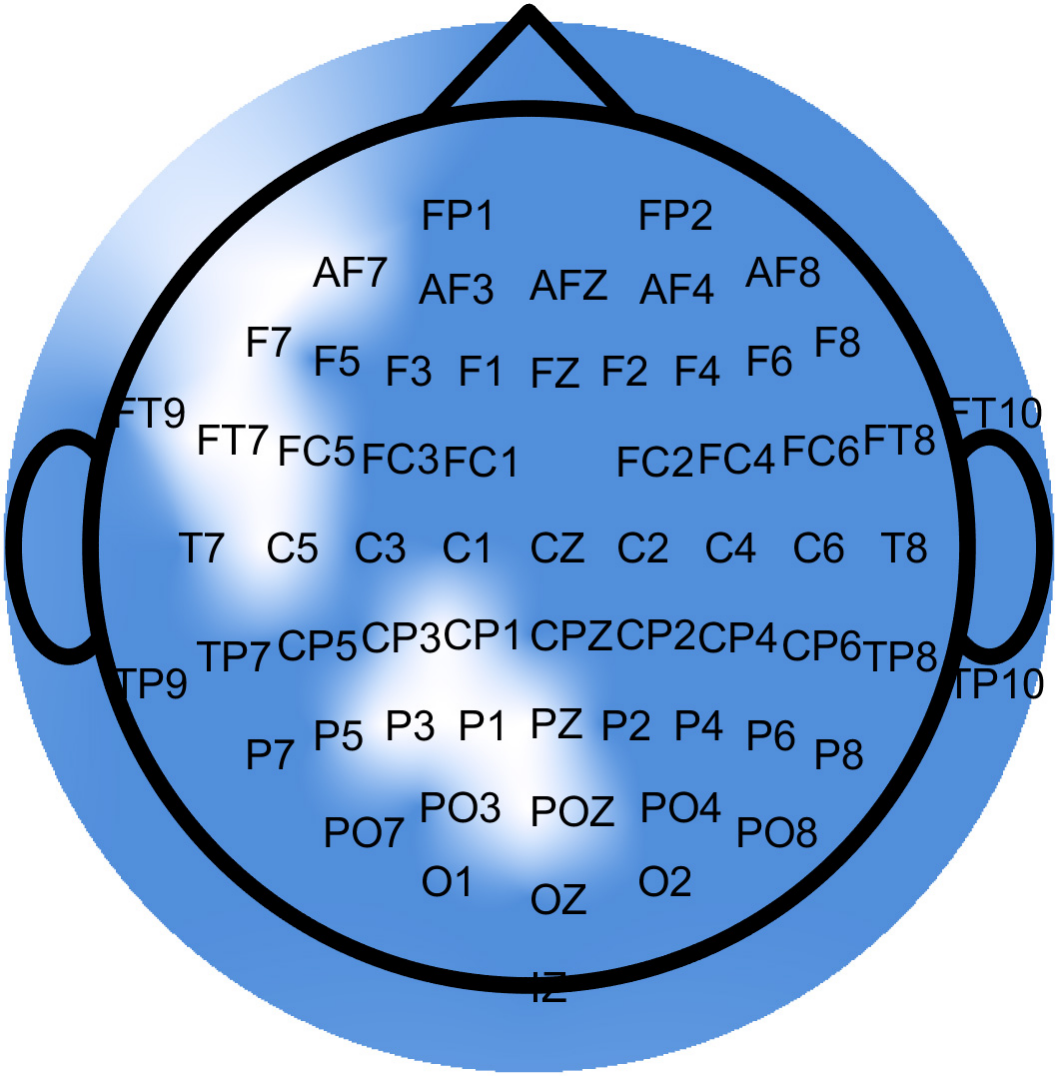
Topographical distribution of resting-state entropy changes following the exergaming intervention. Significant effects were localized over frontal, central, and posterior electrodes marked as white.

**FIGURE 4:**
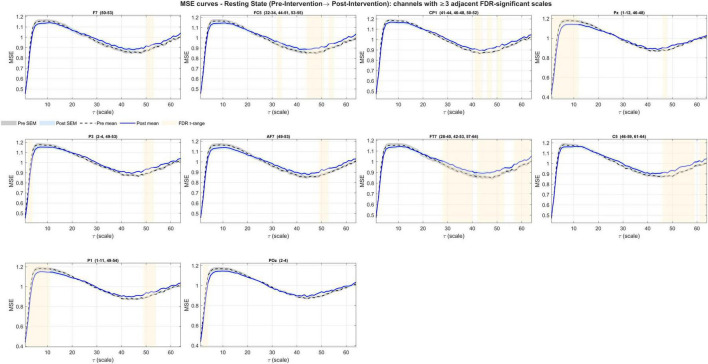
Channels with significant entropy changes at resting-state following the exergaming intervention. A total of ten channels showed significant FDR-corrected bands (shaded ranges) with increases at coarse scales, while P3, P1, Pz, and POz also showed decreases at fine scales.

In pre- to post-intervention resting-state ΔMSE(τ), all channels (63/63) showed greater coherence than expected by chance (lag-1 autocorrelation, right-tailed permutation; *N* = 5,000; all *p* < 1/(N+1) = 0.0002), indicating non-random scale-wise changes. Per-scale paired tests with within-channel FDR control and the ≥ 3 adjacent scales criterion identified contiguous significant bands in 10/63 channels (F7, FC5, CP1, Pz, P3, AF7, FT7, C5, P1, and Poz) (Figure 3), predominantly at mid-to-coarse scales (τ≈28–64) with occasional fine-scale clusters (τ≈1–4). The significant bands are presented for each individual channel in Table 3. The global ΔCI did not reach significance at any channel, consistent with a shape change in the multiscale profile (increases at some scales offset by decreases at others) rather than a uniform shift (Figure 4).

**TABLE 3:** EEG channels with significant false-discovery-rate (FDR)-corrected bands along with the corresponding scale ranges (τ).

Resting (Pre→Post)	Channels	Significant bands	Gameplay (Pre→Mid)	Channels	Significant bands
	F7	50–53		Fz	37–51, 53–55, 57–60, 62–64
	FC5	32–34, 44–51, 53–55		F3	35–37, 39–45
	CP1	41–44, 46–48, 50–52		FC1	40–44, 51–53
	Pz	1–12, 46–48		TP9	35–64
	P3	2–4, 49–53		CP5	1–3, 37–51, 55–57
	AF7	49–53		P7	40–59
	FT7	28–40, 42–53, 57–64		TP10	38–55, 57–59
	C5	46–59, 61–64		Cz	37–64
	P1	1–11, 49–54		C4	1–3, 40–54
	POz	2–4		FC2	30–60, 62–64
Gameplay (Mid→Post)	Channels	Significant bands		F4	44–46, 48–51, 57–60, 62–64
	No significant channels			Fp2	41–44, 53–59
Gameplay (Pre→Post)	Channels	Significant bands		AF7	35–44, 51–55
	F7	51–55		AF3	39–45
	TP9	1–3, 41–44, 60–63		F1	37–48, 53–55, 57–60
	TP10	41–48, 51–53		F5	39–41, 51–53
	Cz	52–55		C1	1–3, 36–48, 50–55, 57–64
	AF7	51–55		C5	49–51, 55–60
	F5	51–55		TP7	40–53, 55–60
Trend (Pre→Mid→Post)	Channels	Significant bands		TP8	51–53
	F7	51–55		C2	1–3, 35–60, 62–64
	TP9	1–3, 41–44, 60–63		FC4	37–40, 42–51, 53–59
	TP10	41–48, 51–53		F6	57–59
	Cz	52–55		AF4	58–60
	AF7	51–55		F2	39–51, 62–64
	F5	51–55			

### 3.3 Gameplay multiscale entropy

**FIGURE 5:**
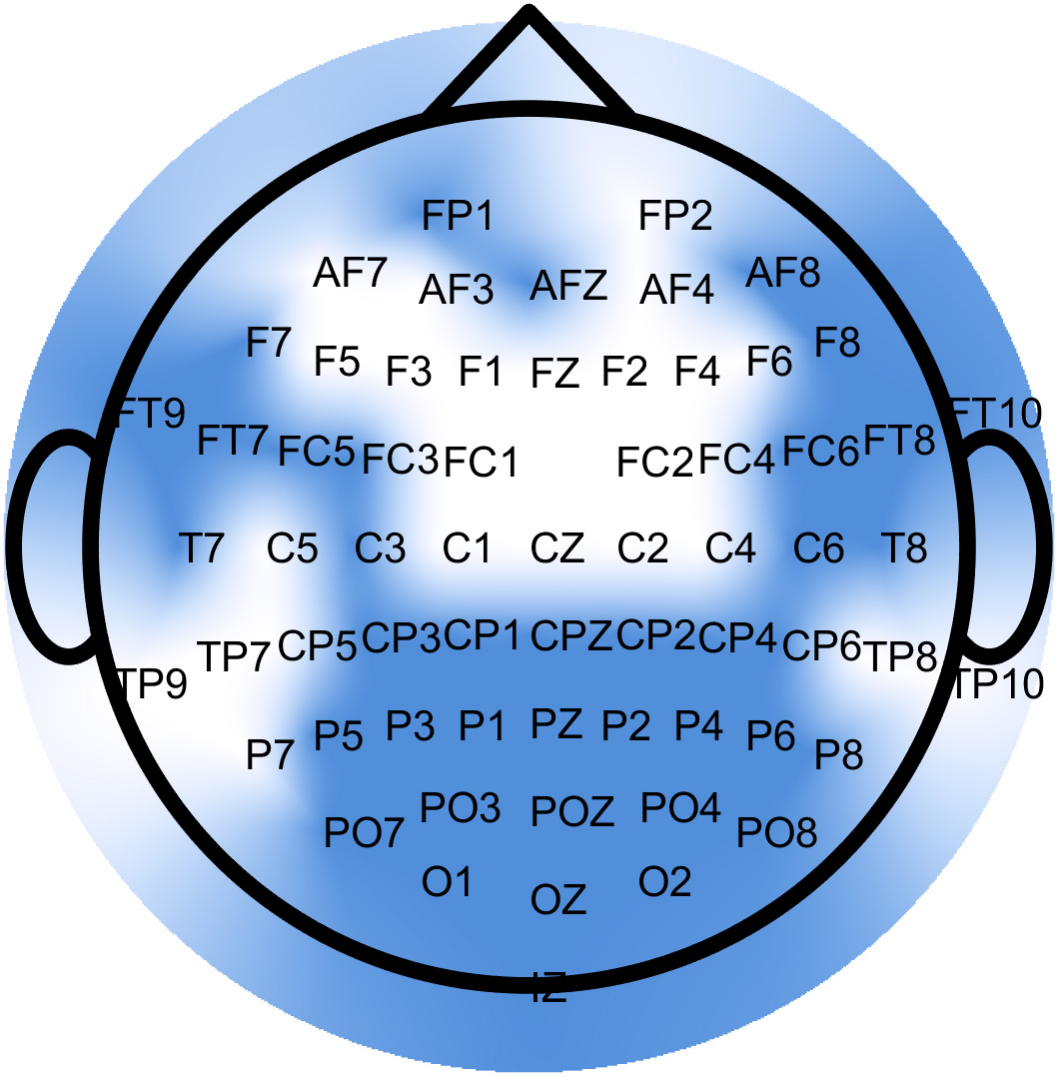
Topographical distribution of gameplay entropy changes from pre- to mid-intervention during gameplay. Significant effects were observed over frontal, central, and posterior electrodes marked as white.

**FIGURE 6:**
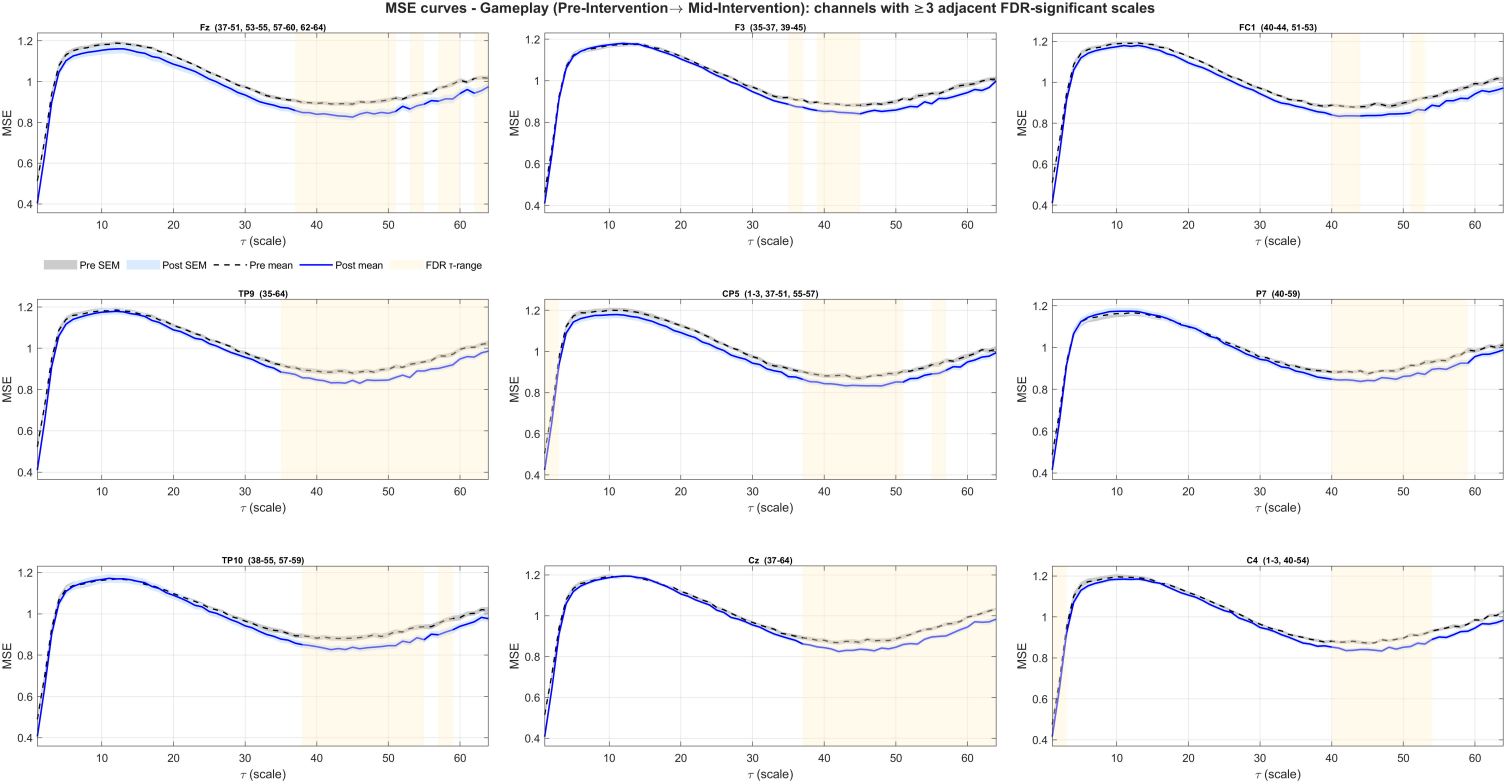
Channels showing significant entropy changes from pre- to mid-intervention during gameplay (first set of 9 channels). A decrease at coarse entropy was observed. Significant FDR-corrected bands are highlighted with shades.

**FIGURE 7:**
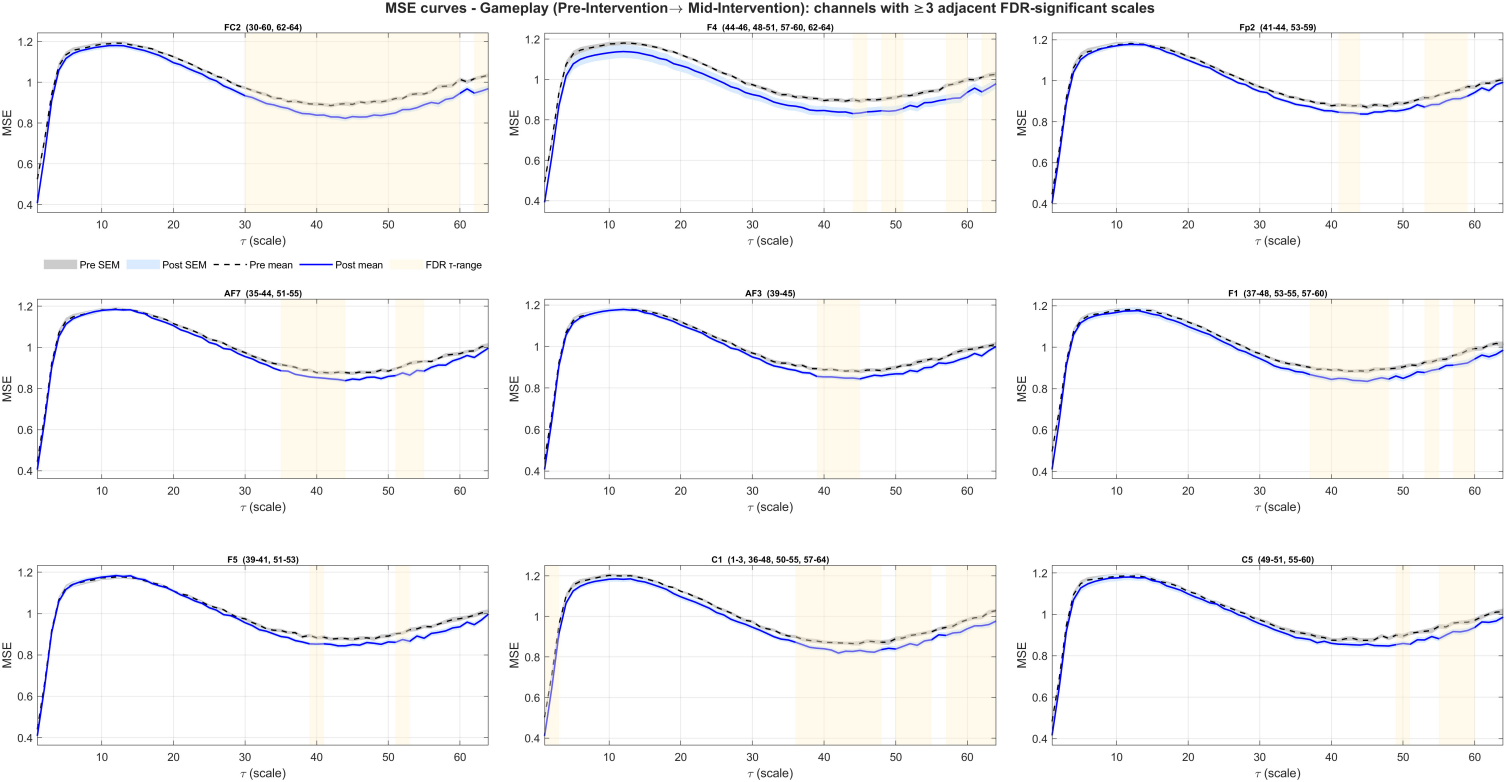
Channels showing significant entropy changes from pre- to mid-intervention during gameplay (the next set of 9 channels). A decrease at coarse entropy was observed. Significant FDR-corrected bands are highlighted with shades.

**FIGURE 8:**
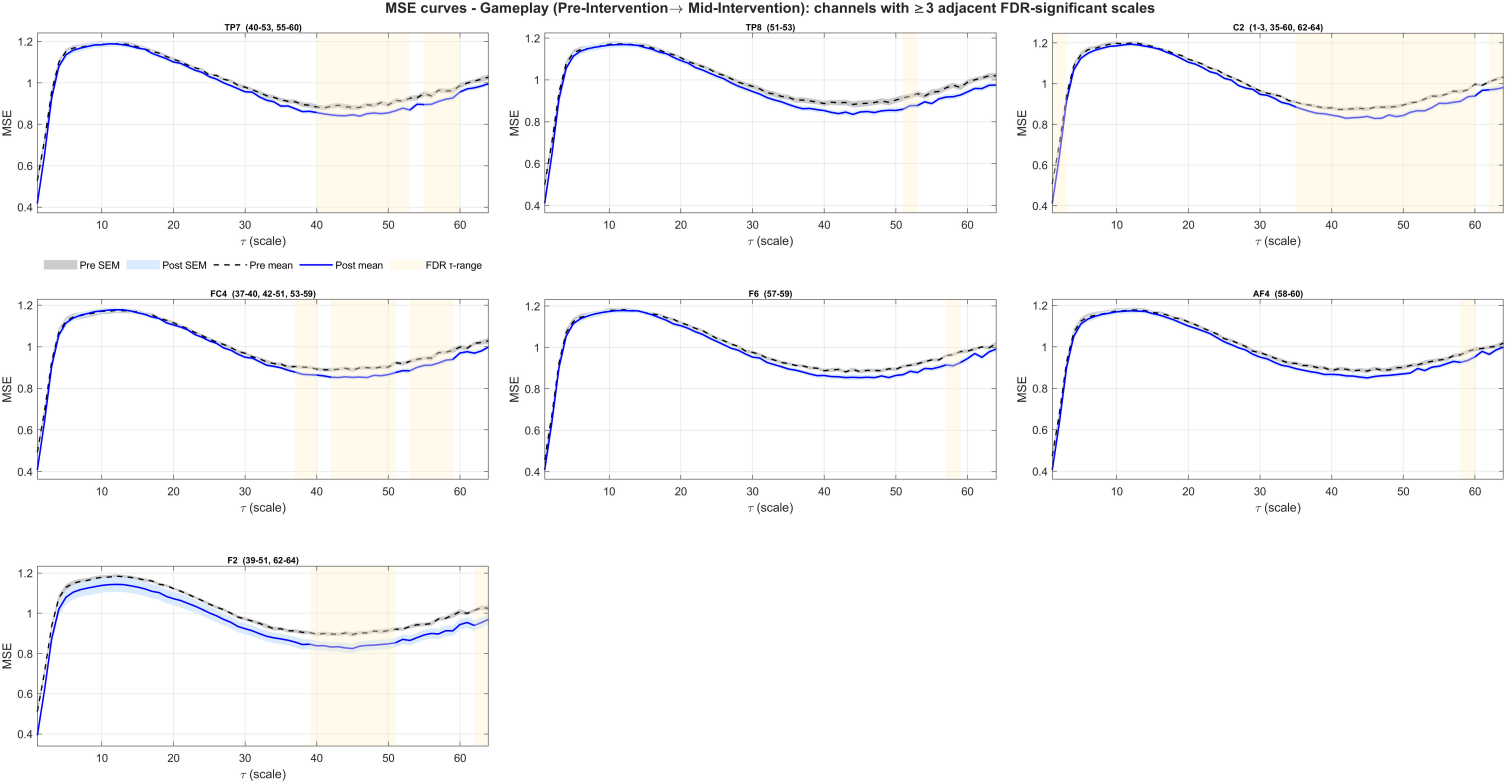
Channels showing significant entropy changes from pre- to mid-intervention during gameplay (the last set of 7 channels). A decrease at coarse entropy was observed. Significant FDR-corrected bands are highlighted with shades.

Pre- to mid-intervention ΔMSE(τ) during gameplay yielded a significant coherence in all channels (63/63; lag-1 autocorrelation, right-tailed permutation; *N* = 5,000; all *p* < 1/(N+1) = 0.0002). Contiguous FDR bands were present in 25/63 channels (Fz, F3, FC1, TP9, CP5, P7, TP10, Cz, C4, FC2, F4, Fp2, AF7, AF3, F1, F5, C1, C5, TP7, TP8, C2, FC4, F6, AF4, F2) (Figure 5). Bands concentrated at coarse scales (typical ranges within τ≈35–64), with brief fine-scale clusters (τ = 1–3) at a subset of channels (Figures 6–8 and Table 3). The global ΔCI was significant in 40/63 channels (two-sided sign-flip permutation) and consistently negative (Supplementary file).

In mid- to post-intervention ΔMSE(τ), coherence remained broad (60/63 channels; lag-1 autocorrelation, right-tailed permutation; *N* = 5,000; all *p* < 1/(N+1) = 0.0002), but no channels showed contiguous FDR bands and only one channel exhibited a positive significant ΔCI (Supplementary file).

**FIGURE 9:**
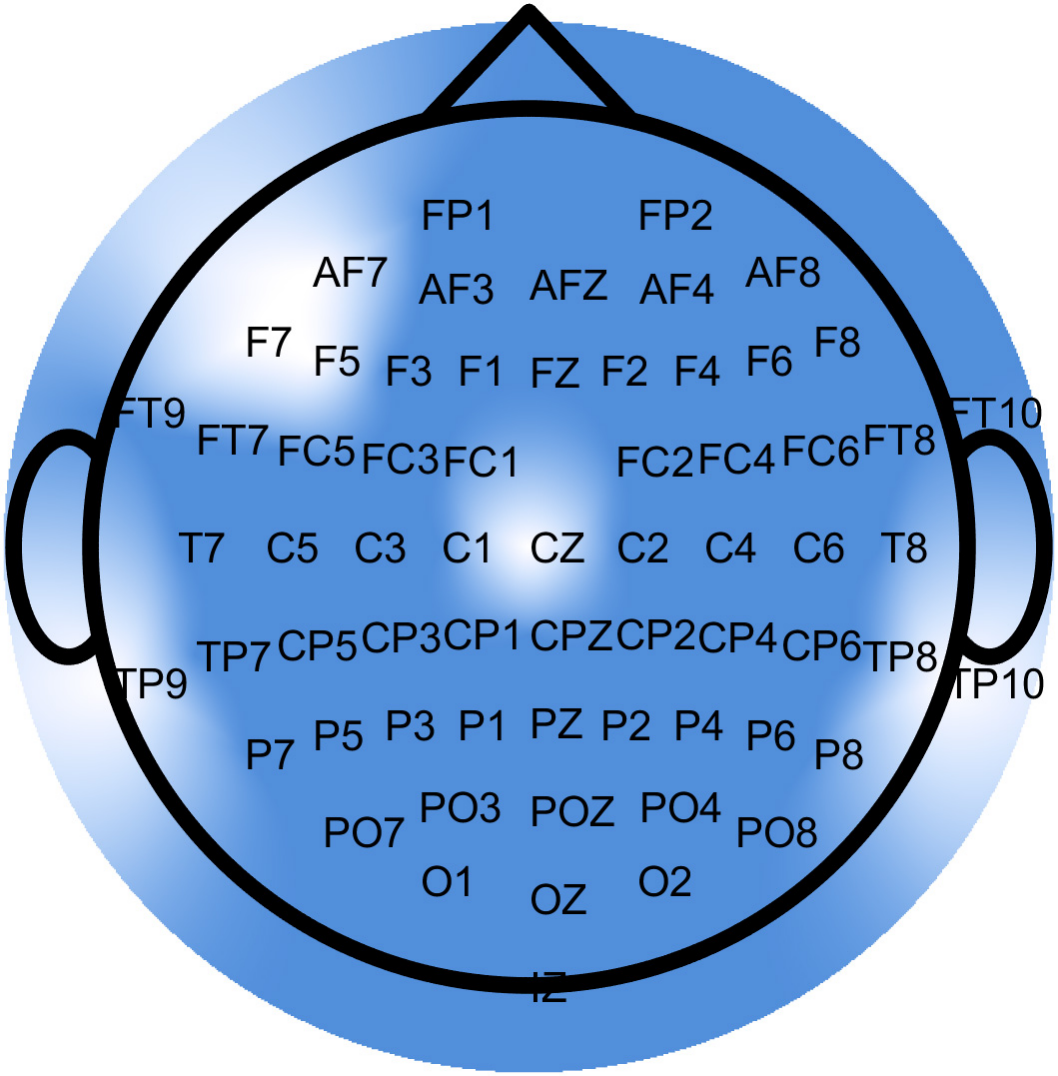
Topographical distribution of net EEG entropy changes from pre- to post-intervention during gameplay. Significant effects were observed over frontal, central, and temporal electrodes marked as white.

**FIGURE 10:**
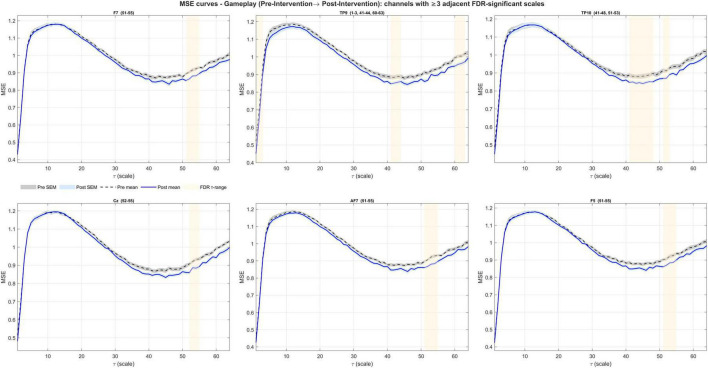
Channels showing significant entropy changes from pre- to post-intervention during gameplay. Six channels exhibited net reductions at coarse scales.

For pre- to post-intervention ΔMSE(τ), coherence was present in 61 channels (lag-1 autocorrelation, right-tailed permutation; N = 5,000; all *p* < 1/(N+1) = 0.0002). Contiguous FDR bands occurred in 6 channels (Figure 9), concentrated at coarse scales (τ≈51–55) with a brief fine-scale run at one channel (τ = 1–3) (Figure 10 and Table 3). The across-scale ΔCI was significant in 28 channels with consistent negative effects (Supplementary file). Furthermore, a within-subject linear trend across sessions (–1, 0, +1 contrast; estimated as Post-Pre for equally spaced sessions) was significant in 28 of these channels (two-sided sign-flip permutation) with uniformly negative slopes, indicating a gradual decrease in coarse-scale complexity across sessions.

## 4 Discussion

The present study investigated the effects of a 4-week exergame intervention on the brain signal complexity of healthy older adults, assessed via MSE of the EEG data at rest and during gameplay. The intervention led to significant improvements in both physical function and in-game performance metrics. Correspondingly, the EEG findings revealed two distinct patterns of change: (1) in resting-state EEG, the intervention induced a significant decrease in fine-scale complexity and an increase in coarse-scale complexity across frontal, central, and posterior brain regions; and (2) during gameplay, brain complexity declined widespread by mid-intervention and then largely stabilized from mid to post, yielding a net reduction in frontal, central and temporal regions by the end of the intervention, concentrated at coarse time scales.

### 4.1 Behavioral findings

The significant improvements observed in the behavioral outcomes confirm the efficacy of exergaming on physical function and skill acquisition. Participants demonstrated an enhanced balance and mobility level, as indicated by increased CBMS scores. This finding is consistent with a large body of literature showing that exergaming is an effective tool for improving physical function and reducing fall risk in older adults (Donath et al., 2016; Fang et al., 2020; Pacheco et al., 2020). Recent meta-analytical evidence showed that mobility gains can be measurable even in independent older adults, suggesting the benefits of exergaming even in high-functioning populations (Suleiman-Martos et al., 2022).

The improvement in the CBMS is particularly noteworthy as it assesses high-level, dynamic balance skills necessary for safe community mobility. The specific demands of the exergames may have contributed to these gains in different domains of the CBMS. The Fox game, requiring continuous and reactive lateral stepping, may have trained motor patterns analogous to tasks such as lateral dodging and maps onto step-training paradigms known to reduce fall risk (Okubo et al., 2017). Similarly, the Puzzle game, which requires controlled mediolateral leaning, may have specifically challenged the ability to shift weight while maintaining a stable base of support—a critical component of dynamic CBMS tasks that is often impaired in older adults (Hatzitaki et al., 2009; de Vries et al., 2014). Furthermore, both games impose a significant cognitive load, demanding visuospatial processing, attention, and decision-making concurrent with motor execution (Müller et al., 2023). This form of integrated cognitive-motor training is particularly effective for older adults, as it mirrors the multitasking demands of real-world mobility (Herold et al., 2018) and specific CBMS items like Walking and Looking (Howe et al., 2006). The simultaneous improvement in all in-game metrics provides evidence of learning and demonstrates that participants successfully adapted to the cognitive and motor demands of the tasks (Müller et al., 2024), a process likely facilitated by the engaging and motivating nature of the exergame environment (Maillot et al., 2012; Stojan and Voelcker-Rehage, 2019).

### 4.2 Resting-state complexity

Following the 4-week intervention, alterations in resting-state brain signal complexity was observed, characterized by a decrease in entropy at fine temporal scales and a concurrent increase at coarse temporal scales across frontal, central, and posterior regions. This specific pattern is particularly pivotal as it opposes the typical trajectory of age-related changes, which involves increased fine-scale and decreased coarse-scale entropy (Sleimen-Malkoun et al., 2015; Wang et al., 2016). The changes observed post-intervention therefore suggest a trend opposite to age-related alterations in brain complexity, reflecting a modulation in the functional architecture toward a more youthful and adaptive profile (McIntosh et al., 2014). The localization of these effects is particularly noteworthy, as frontal, central, and posterior areas are among the regions most consistently reported to show age-related structural alterations (Raz et al., 2005; Fjell and Walhovd, 2010). Moreover, these regions have been documented to demonstrate differences in resting-state complexity between older and younger adults in previous studies, despite differences in resting-state protocols compared with the present study (eyes-open vs. eyes-closed) (Sleimen-Malkoun et al., 2015; Wang et al., 2016).

The increase in coarse-scale entropy is a critical aspect of this finding. Coarse-scale entropy is given to reflect the integration of information across distributed, long-range neural networks (Vakorin et al., 2011; McIntosh et al., 2014). This functional distinction is supported by research linking entropy to other network metrics, which has shown that higher coarse-scale complexity is associated with greater functional connectivity across distributed brain regions (Wang et al., 2018; Ando et al., 2022). With aging, the amount of communication between long-range networks attenuates, leading to increased neural noise (Onoda et al., 2012; Nobukawa et al., 2019). This functional change signifies a shift toward more localized and segregated processing, which impairs the brain’s capacity for flexible information processing (Sleimen-Malkoun et al., 2015; Wang et al., 2016). Correspondingly, higher coarse-scale complexity in older adults is positively correlated with superior cognitive performance (Ueno et al., 2015; Iinuma et al., 2022). This link is further supported by cross-sectional evidence showing that physically active older adults exhibit greater coarse-scale entropy than their sedentary counterparts, which is interpreted as a marker of greater neural adaptability (Wang et al., 2014). The observed increase in coarse-scale entropy thus indicates that the exergame intervention may enhance the brain’s capacity for long-range communication, a key feature of a healthy, adaptive neural system. Conversely, the concurrent decrease in fine-scale entropy suggests a reduction in local, and potentially noisy, neural processing (Ando et al., 2022). This dual effect—enhancing global integration while reducing local segregation—points to a beneficial short-term adaptation of intrinsic brain dynamics, underscoring that the intervention did not simply increase or decrease overall complexity, but rather modulated its profile across time scales. The continuous multi-domain integration inherent to the exergame may have driven this adaptation, including processing and updating dynamic visuospatial information in real time, adjusting motor behavior accordingly and developing strategies to fulfill increasing demands, all of which are processes shown to modulate complex, long-range neural networks (Lungarella and Sporns, 2006; Monteblanco Cavalcante et al., 2021; Henrique et al., 2023).

### 4.3 Complexity during gameplay

In contrast to the resting-state results, brain signal complexity during gameplay showed a pronounced decrease, particularly from pre- to mid-intervention. This divergence underscores that brain complexity is not a static property but rather state-dependent, reflecting fundamentally different modes of information processing during task engagement versus rest (Gu et al., 2018; Kaur et al., 2019; Gu et al., 2022). The observed decrease in complexity is best interpreted as a neurodynamic signature of learning and the development of automaticity, which is particularly relevant for older adults who often exhibit a reduced capacity for adaptability and an increased reliance on cognitive resources to guide performance (Voelcker-Rehage, 2008; Seidler et al., 2010). From an information-theoretical perspective, a novel task environment creates high uncertainty, requiring extensive neural computations that manifest as high signal complexity (Beni et al., 2025). With practice, as the tasks becomes familiar, this uncertainty diminishes, leading to a quantifiable decrease in brain signal complexity (Cnudde et al., 2021; Parbat and Chakraborty, 2021; Beni et al., 2025).

This reduction in complexity during gameplay aligns with the neural efficiency hypothesis, which posits that as new skills are acquired, the brain requires fewer neural resources to achieve the same or superior level of performance (Neubauer and Fink, 2009). Importantly, the temporal trajectory of these changes—marked by a decline from pre- to mid-intervention followed by relative stabilization—suggests that neural efficiency gains emerge rapidly during early practice and then plateau as the system settles into an optimized operating regime (Anderson, 1982; Chein and Schneider, 2005; Kelly and Garavan, 2005). During skill acquisition, neural dynamics typically transit from exploratory, variable states to more stabilized, task-tuned regimes, resulting in reduced information processing and lower signal complexity (Wang et al., 2018; Kaur et al., 2019; Parbat and Chakraborty, 2021; Gu et al., 2022). This interpretation is supported by evidence from another task-based study (Cnudde et al., 2021), where reductions in coarse-scale entropy have been associated with an attenuated need for transition between states and a more simplified neural response (Chein and Schneider, 2005; Grill-Spector et al., 2006). Because coarse scales capture slow, integrative fluctuations shaped by long-range network communication, reductions in this range likely indicate a streamlining of large-scale coordination as the brain settles into a more efficient, task-specific operating mode (He, 2014; McIntosh et al., 2014; Kosciessa et al., 2020). Furthermore, the localization of reduced entropy over frontal, central, and temporal regions aligns with the spatial distribution of key functional networks critical to develop strategies while executing motor tasks (Henschke and Pakan, 2023). Specifically, the frontoparietal attentional, sensorimotor and salience networks are shown to modulate learning by potentially streamlining attentional, sensorimotor and visuomotor processes (Menon and Uddin, 2010; Penalver-Andres et al., 2021; Chen et al., 2025; Rezaei et al., 2025). The reductions in the coarse complexity of these regions align with the adaptation of long-range communication, refining goal-maintenance systems to achieve a more-steady state during tasks, characterized by a more automatized executive control, enhanced sensorimotor processing and visuomotor integration (Kelly and Garavan, 2005; Steele and Penhune, 2010; Babiloni et al., 2016). In the present study, this neural adaptation parallels significant improvements in in-game metrics across three time points, indicating that refinements in long-range communication coincide with a more effective game performance. Within the exergaming context, such streamlining may enable older adults to engage in more automatic responses, thereby alleviating the cognitive demands typically required to sustain dual performance (Seidler et al., 2010; Herold et al., 2018).

Overall, the present study provides converging evidence that exergaming may have the potential to alter brain complexity in older adults both at rest and during tasks. At rest, the post-intervention complexity pattern was altered in a direction that contrasts with typical age-related trends, resembling characteristics more commonly observed in younger adults, typically expressed as decreased fine-scale and increased coarse-scale entropy. During gameplay, complexity reductions followed a rapid–stabilizing trajectory, reflecting early neural efficiency gains and the automatization of cognitive-motor control. Together with significant improvements in balance, mobility, and in-game performance, these findings highlight exergaming as a potential, prophylactic intervention that not only enhances physical function but may also promote a healthy brain complexity profile. By targeting the interplay between motor and cognitive domains, exergames offer a practical, engaging, and scalable approach to mitigate age-related decline and support healthy brain aging.

### 4.4 Methodological limitations and perspectives

Several methodological limitations should be acknowledged when interpreting and translating the current findings. First, the study was conducted as a short-term, 4-week intervention without follow-up assessments, precluding conclusions about the long-term stability of the observed effects (Lampit et al., 2014; Ngandu et al., 2015). Second, the absence of a control group limits causal inference, as non-specific factors such as repeated testing cannot be excluded. Future studies should incorporate both passive controls and active controls (alternative physical or cognitive training) to disentangle intervention-specific effects (Boot et al., 2013; Stanmore et al., 2017). Third, no post-intervention cognitive tests were administered, which restricts conclusions about whether exergaming induced detectable cognitive improvements (Gheysen et al., 2018). Still, improvements in in-game performance metrics across the intervention, together with previously reported increases in cognitive engagement in this dataset (Müller et al., 2025), suggest that learning-related cognitive gains may have accompanied the neural changes observed in the present study.

In addition, the sample consisted of relatively healthy, well-functioning older adults, which enhances internal validity but limits generalizability to more diverse or clinical cohorts (Zhao et al., 2020). Gameplay analyses were restricted to the Fox game to ensure standardization, potentially underestimating the broader spectrum of exergaming-induced adaptations. As another point, although our preprocessing pipeline has been previously used in MSE analysis with mobile EEG data (Piskin et al., 2025), preprocessing operations like filtering can influence entropy values, and further work is needed to establish best practices (Puglia et al., 2022). Finally, the interpretation of entropy in mobile tasks remains tentative, as most evidence stems from cognitive paradigms (Sleimen-Malkoun et al., 2015; Cnudde et al., 2021).

Looking forward, future studies should employ longer intervention periods and include both active and passive control groups to more clearly dissociate intervention-related effects from test–retest changes. Incorporating follow-up assessments and standardized cognitive outcomes would help determine the durability and behavioral relevance of the observed neural adaptations. In addition, studies that systematically manipulate game characteristics and intervention frequencies, examine the efficacy of exergaming in cohorts with different functional levels, and adopt entropy metrics as outcomes could deepen the understanding of brain complexity in aging context and support the development of both prophylactic and therapeutic exergaming interventions.

## Data Availability

The raw data supporting the conclusions of this article will be made available by the authors, without undue reservation.
