# Supplementary material for: Rewiring the aging brain: exergaming modulates brain complexity in older adults
Source: Front Aging Neurosci. 2026 Jan 12;17:1748274. doi: 10.3389/fnagi.2025.1748274 (PMC12832828; doi:10.3389/fnagi.2025.1748274)
Supplement: Supplementary file 1 [file Data_Sheet_1.pdf]

Paper Section / Topic	Item No	Descriptor	Reported?
**Title and Abstract**			
Title and Abstract	1	Information on how units were allocated to interventions	N/A
		Structured abstract recommended	Yes
		Information on target population or study sample	Yes
**Introduction**			
Background	2	Scientific background and explanation of rationale	Yes
		Theories used in designing behavioral interventions	Yes
**Methods**			
Participants	3	Eligibility criteria for participants, including criteria at different levels in recruitment/sampling plan (e.g., cities, clinics, subjects)	Yes
		Method of recruitment (e.g., referral, self-selection), including the sampling method if a systematic sampling plan was implemented	Yes
		Recruitment setting	Yes
		Settings and locations where the data were collected	Yes
Interventions	4	Details of the interventions intended for each study condition and how and when they were actually administered, specifically including:	Yes
		• Content: what was given?	Yes
		• Delivery method: how was the content given?	Yes
		• Unit of delivery: how were the subjects grouped during delivery?	N/A
		• Deliverer: who delivered the intervention?	Yes
		• Setting: where was the intervention delivered?	Yes
		• Exposure quantity and duration: how many sessions/episodes/events were intended? How long were they to last?	Yes
		• Time span: how long was it intended to take to deliver the intervention to each unit?	Yes
		• Activities to increase compliance or adherence (e.g., incentives)	N/A
Objectives	5	Specific objectives and hypotheses	Yes
Outcomes	6	Clearly defined primary and secondary outcome measures	Yes

		Methods used to collect data and any methods used to enhance the quality of measurements	Yes
		Information on validated instruments such as psychometric and biometric properties	Yes
Sample Size	7	How sample size was determined and, when applicable, explanation of any interim analyses and stopping rules	N/A
Assignment Method	8	Unit of assignment (the unit being assigned to study condition, e.g., individual, group, community)	N/A
		Method used to assign units to study conditions, including details of any restriction (e.g., blocking, stratification, minimization)	N/A
		Inclusion of aspects employed to help minimize potential bias induced due to non-randomization (e.g., matching)	N/A
Blinding (masking)	9	Whether or not participants, those administering the interventions, and those assessing the outcomes were blinded to study condition assignment; if so, statement regarding how the blinding was accomplished and assessed	N/A
Unit of Analysis	10	Description of the smallest unit that is being analyzed to assess intervention effects (e.g., individual, group, or community)	Yes
		If the unit of analysis differs from the unit of assignment, the analytical method used to account for this (e.g., multilevel analysis)	N/A
Statistical Methods	11	Statistical methods used to compare study groups for primary outcome(s), including complex methods of correlated data	Yes
		Statistical methods used for additional analyses, such as subgroup analyses and adjusted analysis	Yes
		Methods for imputing missing data, if used	N/A
		Statistical software or programs used	Yes

Results			
Participant flow	12	Flow of participants through each stage of the study: enrollment, assignment, allocation, intervention exposure, follow-up, analysis (diagram recommended)	Yes
		Enrollment: numbers screened, eligible, not eligible, declined, and enrolled	No
		Assignment: numbers assigned to a study condition	N/A
		Allocation and exposure: number assigned to each condition and number who received each intervention	N/A
		Follow-up: number who completed or did not complete follow-up (lost to follow-up) by study condition	N/A
		Analysis: number included in or excluded from the main analysis, by study condition	N/A
		Description of protocol deviations from study as planned, along with reasons	N/A
Recruitment	13	Dates defining the periods of recruitment and follow-up	N/A
Baseline Data	14	Baseline demographic and clinical characteristics of participants in each study condition	Yes
		Baseline characteristics relevant to specific disease prevention research	N/A
		Baseline comparisons of those lost to follow-up and those retained	N/A
		Comparison between study population at baseline and target population of interest	N/A
Baseline Equivalence	15	Data on study group equivalence at baseline and statistical methods used to control for baseline differences	N/A
Numbers Analyzed	16	Number of participants (denominator) included in each analysis for each study condition; results in absolute numbers when feasible	Yes
		Indication of "intention to treat" analysis or description of how non-compliers were treated	N/A
Outcomes and Estimation	17	For each outcome, summary of results for each condition, estimated effect size, and confidence interval	Yes
		Inclusion of null and negative findings	Yes
		Inclusion of results from testing pre-specified causal pathways	N/A

Ancillary Analyses	18	Summary of other analyses performed (subgroup/restricted), indicating if pre-specified or exploratory	N/A
Adverse Events	19	Summary of all important adverse events or unintended effects in each study condition	N/A
**Discussion**			
Interpretation	20	Interpretation of results, taking into account hypotheses, potential bias, imprecision, and other limitations	Yes
		Discussion of results taking into account causal pathways or alternative mechanisms	Yes
		Discussion of success/barriers to implementation and fidelity of implementation	Yes
		Discussion of research, programmatic, or policy implications	Yes
Generalizability	21	External validity taking into account population, intervention characteristics, follow-up, compliance, and settings	Yes
Overall Evidence	22	General interpretation of results in the context of current evidence and theory	Yes
